# Biomechanical analysis of maxillary incisor intrusion with clear aligners under different periodontal conditions: a finite element analysis

**DOI:** 10.3389/fbioe.2026.1921947

**Published:** 2026-09-23

**Authors:** Qiuying Li, Yidan Zhang, Bowen Xu, Linna Bai, Kai Yang

**Affiliations:** Department of Orthodontics, Beijing Stomatological Hospital, Capital Medical University, Beijing, China

**Keywords:** clear aligner, finite element analysis, maxillary incisor intrusion, orthodontic, periodontal condition

## Abstract

**Objectives:**

To investigate the biomechanical effects and determine the optimal increment for maxillary incisor intrusion using clear aligners across three periodontal conditions via finite element analysis (FEA).

**Materials and methods:**

Nine FE models were created in a 3 × 3 design simulating three periodontal states (M1: normal bone level; M2: one-third alveolar bone loss; M3: one-half alveolar bone loss) combined with three intrusion increments (0.10, 0.15, and 0.20 mm). Anterior tooth displacement, root von Mises stress, and periodontal ligament (PDL) maximum compressive stress were analyzed.

**Results:**

Tooth displacement and mechanical stresses escalated with greater bone loss and larger increments. Central incisors experienced more pronounced tipping, whereas lateral incisors achieved greater vertical intrusion. Consequently, lateral incisors endured higher stresses; their PDL stress was approximately double that of central incisors. While central incisor intrusion efficiency increased proportionally with increment, lateral incisors in M2 and M3 states demonstrated higher efficiency at the 0.15-mm increment (peaking at 92.0%) than at 0.20 mm. In the M3 state, increasing the increment from 0.10 to 0.20 mm sharply amplified lateral incisor root stress (2.96–8.18 MPa) and PDL stress (0.056–0.114 MPa).

**Conclusion:**

Clear aligners produce maxillary incisor intrusion with concurrent tipping. Lateral incisors show higher intrusion efficiency but endure greater mechanical stress than central incisors. Alveolar bone loss exacerbates both tipping tendency and stress. For patients with alveolar bone loss exceeding one-third, the intrusion increment is recommended to be limited to 0.15 mm.

## 1 Introduction

Periodontitis is a multifaceted chronic inflammatory condition that drives the gradual degradation of the periodontium, affecting more than 50% of adults worldwide (Kassebaum et al., 2017). Such deterioration frequently results in pathologic drifting, incisor flaring, and traumatic occlusion. Ultimately, these complications trigger tooth loss, detrimentally affecting oral function, facial esthetics, and overall quality of life (Herrera et al., 2022). In recent years, pathological tooth migration resulting from periodontitis has increased the demand for orthodontic treatment among adult patients (Zasčiurinskienė et al., 2023).

Extrusion and flaring of maxillary incisors are among the most common chief complaints of periodontitis patients seeking orthodontic treatment. To address this, treatment goals typically include space closure, retraction of the flared incisors, and the reduction of anterior overbite and overjet, which necessitate the orthodontic intrusion of the extruded teeth (Zhong et al., 2025). Nevertheless, applying intrusive mechanics to a reduced periodontium remains a subject of ongoing debate. First, orthodontic intrusion risks translocating supragingival biofilm into the subgingival space, thereby aggravating alveolar bone loss and triggering the formation of intrabony defects (Ericsson and Thilander, 1978; Melsen et al., 1988). Second, poorly controlled intrusive forces can induce periodontal ischemia and root resorption (Melsen et al., 1989). Notwithstanding these risks, extensive evidence indicates that meticulous inflammatory management combined with the application of low-magnitude forces allows orthodontic intrusion to improve periodontal parameters, particularly within the anterior region (Cao et al., 2015; Zasčiurinskienė et al., 2018).

For patients with periodontitis, clear aligners (CAs) offer superior oral hygiene maintenance relative to fixed appliances, primarily owing to their removable nature (Jiang et al., 2018; Liu et al., 2024). However, the instantaneous stress applied upon initial CA insertion can be 50 to 500 times greater than that of fixed appliances (Ma and Li, 2021). Consequently, the application of CAs in periodontitis patients warrants further investigation. To date, there is a paucity of literature addressing CA therapy within this specific cohort, with the majority being case reports (Lee et al., 2011; Zhang et al., 2022). While Ma et al. used finite element analysis (FEA) to explore the optimal increment for mandibular incisor intrusion across various periodontal conditions, research focusing on maxillary incisor intrusion remains scarce (Ma and Li, 2021). The biomechanical characteristics of this movement and the appropriate activation increments under different periodontal states have yet to be fully elucidated.

Three-dimensional (3D) FEA is a well-established biomechanical research method widely used within the domain of orthodontic biomechanics (Gao et al., 2024; Li et al., 2024; Xiao et al., 2025). This study aims to simulate the intrusion of maxillary incisors using CAs under three distinct periodontal conditions via 3D FEA. By conducting a comprehensive analysis and determining the optimal intrusive increment, this research seeks to provide biomechanical insights into the clinical practice of CA therapy for patients with periodontitis.

## 2 Materials and methods

This study was approved by the Ethics Committee of Beijing Stomatological Hospital, Capital Medical University (CMUSH-IRB-KJ-PJ-2025-21), and written informed consent was collected from all subjects involved in the research.

### 2.1 Experimental design and grouping

A total of nine models were designed based on a 3 × 3 combination of periodontal conditions and intrusion increments.

1. Periodontal Conditions (as defined by the 2018 consensus classification of periodontal and peri-implant diseases and conditions (Caton et al., 2018)):
  - M1: Stage I periodontitis (Normal alveolar bone level)
  - M2: Stage II periodontitis (One-third alveolar bone loss)
  - M3: Stage III periodontitis (One-half alveolar bone loss)
2. Intrusion Increments:

For each of the above conditions, three intrusion increments (0.10 mm, 0.15 mm, and 0.20 mm) were applied to the maxillary incisors.

### 2.2 Establishment of the 3D FE models

Cone-beam computed tomography (CBCT) data were acquired from an adult male participant for model construction. The voxel size of the CBCT images was 0.2 mm. The subject exhibited an intact permanent dentition, excluding the third molars, with well-preserved dental anatomy, no alveolar bone loss, and a normal labial inclination of the maxillary anterior teeth (U1-SN angle = 102°).

#### 2.2.1 Modeling of the maxilla, periodontal ligament and maxillary dentition

The CBCT datasets (DICOM format) were loaded into Mimics 15.01 (Materialise NV, Belgium) to segment the maxillary dentition and maxilla (M1) based on grayscale thresholds, exporting them as STL files. Following initial surface smoothing in Geomagic Studio 2013 (3D Systems, United States), the dental geometry was transferred to Pro/E 5.0 (PTC, United States), where the periodontal ligament (PDL) was constructed by applying a uniform 0.20-mm outward offset to the root surfaces (Hong et al., 2021). These components were then assembled and surface-fitted in Geomagic to form a watertight 3D model of Stage I periodontitis. The finalized 3D geometries of the maxilla, PDL, and maxillary dentition were successfully established (Figure 1A).

**FIGURE 1:**
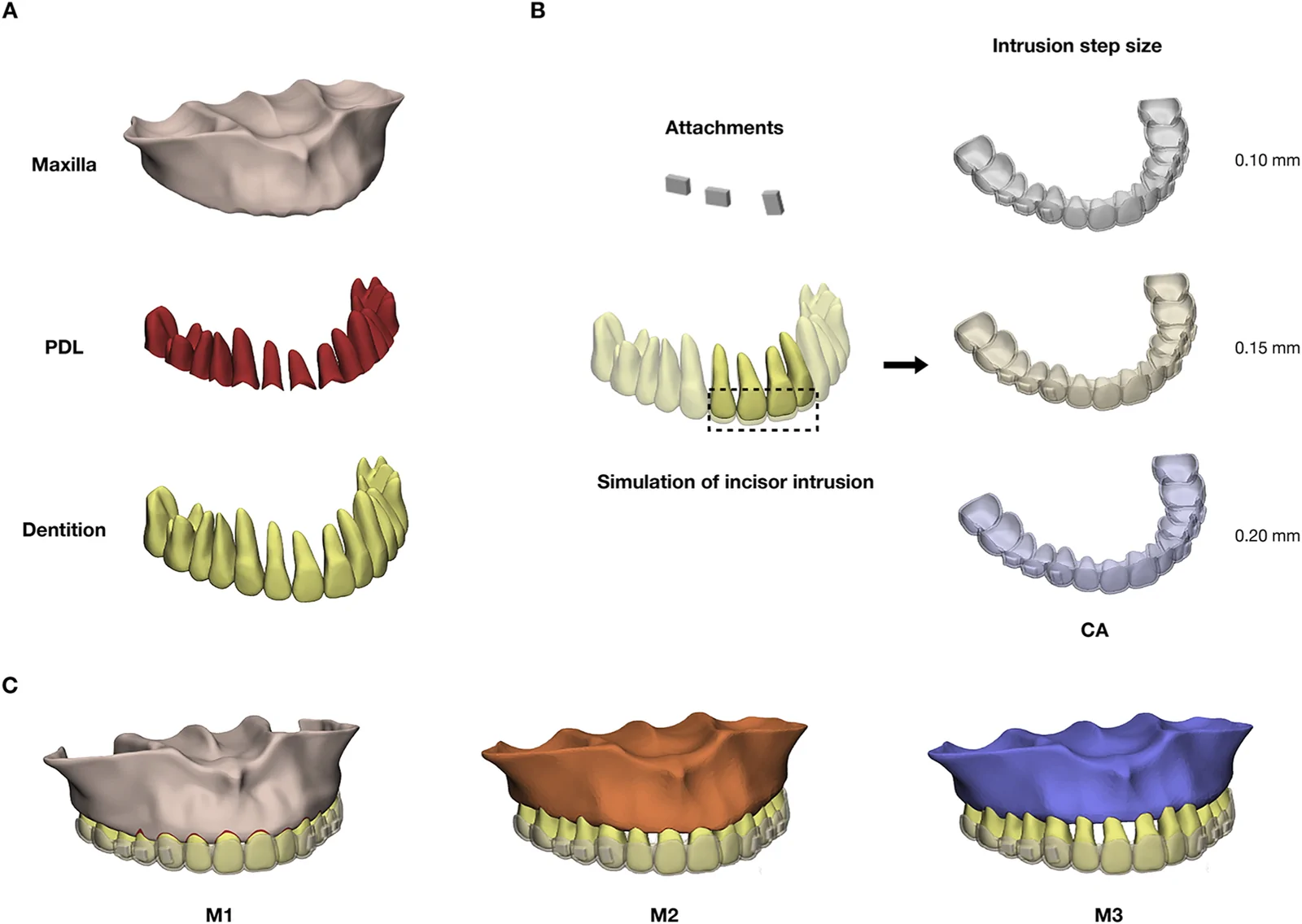
Finite element (FE) models and group design. **(A)**Models of the maxilla, periodontal ligament (PDL), and maxillary dentition reconstructed from volunteer CBCT data. **(B)**Modeling of attachments and clear aligners (CAs), including the design of intrusion increments. **(C)** Assembled FE models under three different periodontal conditions. M1: Normal alveolar bone level; M2: One-third alveolar bone loss; M3: One-half alveolar bone loss.

For M2 and M3 states, the maxilla (M1) was modified in Pro/E by horizontally reducing the alveolar bone height by one-third and one-half, respectively. The corresponding PDL layers were similarly created by a 0.2-mm expansion of the intraosseous root surfaces. Using the same assembly protocol, the 3D models for Stage II and Stage III periodontitis were completed.

#### 2.2.2 Modeling of attachments and CAs

The attachments and CA models were both generated in Pro/E. Vertical rectangular attachments were designed on the labial surfaces of the bilateral canines, while horizontal rectangular attachments were positioned on the buccal surfaces of the premolars. All attachments were positioned at the center of the clinical crowns, with dimensions of 3 × 2 × 1 mm.

To simulate the intervention, the four maxillary incisors were virtually intruded along their long axes according to the three designed increments (0.10 mm, 0.15 mm, and 0.20 mm). To ensure proper intrusion, sufficient space was reserved between the anterior teeth. The aligner trimline was set 2-mm coronal to the cementoenamel junction (CEJ). Using this boundary, the clear aligner models corresponding to each increment were generated by outwardly offsetting the crown surfaces by a constant 0.75-mm thickness (Fan et al., 2022). The geometric models of the attachments and the CAs featuring three different intrusion increments are illustrated in Figure 1B.

All the generated solid models were transferred to Hypermesh 13.0 (Altair Engineering, Inc., United States) for meshing. Tetrahedral elements were used for the maxilla, teeth, attachments, and aligners, whereas wedge elements were employed for the PDL, given its thin-layer structure, to accurately capture its deformation. This study performed a mesh convergence test using a fluctuation amplitude within ±5% in maxilla von Mises stress as the acceptance criterion for accuracy. Under different loading conditions, results computed with varying mesh densities were compared to determine the minimum number of elements needed for reliable outcomes. Comparisons were conducted using maximum mesh sizes of 0.3, 0.2, 0.16, and 0.12 mm, and stable results were obtained when the element size was controlled within the range of 0.005–0.15 mm. Table 1 summarizes the node and element counts for the individual components. Assembly and finalization of the 3D FE models were performed in Abaqus 6.14 (Dassault Systèmes Corp., France). Figure 1C illustrates the assembled models with three different periodontal conditions.

**TABLE 1:** Material properties.

Component	Young’s modulus/MPa	Poisson’s ratio
Maxilla	1.37 × 10^4^	0.30
PDL	0.67	0.45
Teeth	1.96 × 10^4^	0.30
Attachment	12.5 × 10^3^	0.36
CA	842	0.36

PDL: periodontal ligament. CA: clear aligner.

### 2.3 Material properties and boundary conditions

Following prior reports, Maxilla, teeth, attachments and CAs were characterized as homogeneous, isotropic, and linearly elastic (Liu et al., 2022). Because the choice between linear and nonlinear elastic properties for the PDL did not influence long-term orthodontic tooth movement, the PDL was modeled as a linear elastic material to optimize computational efficiency while maintaining accuracy (Kojima and Fukui, 2012). The material properties of all components are shown in Table 2 (Gomez et al., 2015; Fang et al., 2020; Liu et al., 2022).

**TABLE 2:** Element and node numbers for each component of the FE model.

Component	M1		M2		M3	
	Elements	Nodes	Elements	Nodes	Elements	Nodes
Maxilla	358,873	74,341	127,768	27,607	120,809	26,125
PDL	61,623	19,995	32,205	10,914	33,596	11,268
Teeth	70,856	20,656	70,856	20,656	70,856	20,656
Attachment	500	249	500	249	500	249
CA	48,416	15,493	48,416	15,493	48,416	15,493

FE: finite element. PDL: periodontal ligament. CA: clear aligner.

Bonded contacts were assigned between the maxilla and the PDL, the PDL and the teeth, as well as the teeth and the attachments. Frictional contact was defined between the CAs and the surfaces of the teeth and attachments, with a friction coefficient (μ) of 0.2 (Ramalho and Antunes, 2007).

### 2.4 Establishment of the coordinate system

This study employed two coordinate systems. A global reference frame was anchored to the maxilla, with the X-, Y-, and Z-axes aligned along the mesiodistal, labiolingual, and vertical directions, respectively. Additionally, a local reference frame was assigned to each anterior tooth to facilitate displacement analysis. The local coordinate origin was set at the midpoint of the incisal edge for incisors and at the cusp tip for canines. The X-, Y-, and Z-axes represented the mesiodistal, labiolingual, and vertical directions, with positive values indicating distal, lingual, and intrusive movements, respectively.

### 2.5 Biomechanical analysis and outcomes

Owing to the bilateral symmetry of the maxillary dentition, only the right central incisor, lateral incisor, and canine were analyzed as representative teeth.

Displacement evaluation of teeth was conducted at two levels: the maxillary dentition and individual anterior teeth in three dimensions. To characterize displacement patterns in greater detail, crown and root displacements were assessed separately. Crown displacement was tracked at the previously defined local origin of each tooth, while root displacement was monitored at the corresponding apex. Furthermore, the incisor intrusion efficiency was calculated to assess the absolute intrusion along their long axes. This efficiency was defined as the ratio of the achieved intrusion along the Z-axis of the local coordinate system to the designed intrusion amount (0.10 mm, 0.15 mm, and 0.20 mm). Besides, the displacement of CAs was also evaluated. Two stress metrics were examined for each incisor: von Mises stress in the root and maximum compressive sress in the PDL.

## 3 Results

The resultant displacement contours of the dentition across all nine models are shown in Figure 2. During the intrusion of the four maxillary incisors, displacement was primarily localized to the six maxillary anterior teeth. Under different periodontal conditions, anterior tooth displacement increased progressively with larger intrusion increments. Similarly, for a given intrusion increment, anterior displacement also increased gradually with greater alveolar bone loss. The greatest displacement of the maxillary anterior teeth occurred in the M3 group with a 0.2-mm intrusion increment.

**FIGURE 2:**
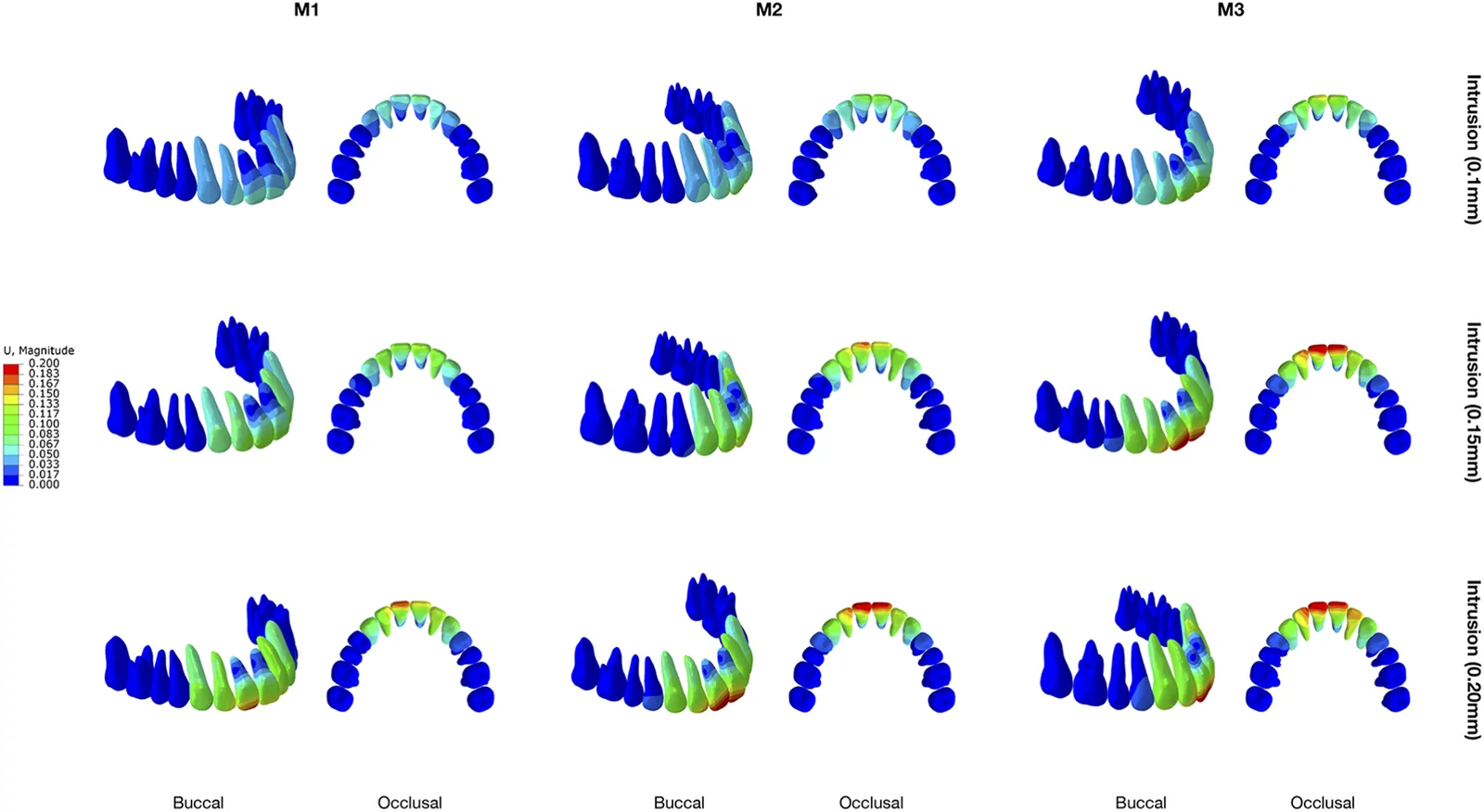
Displacement contour plot of the maxillary dentition. M1: Normal alveolar bone level; M2: One-third alveolar bone loss; M3: One-half alveolar bone loss.

The 3D displacement trends of individual teeth were consistent across all nine models. Vertically, intrusive movement occurred in the central and lateral incisors, while extrusive movement was observed in the canines. Labiolingually, both the incisors and canines exhibited tipping, characterized by lingual crown displacement and labial root displacement. Mesiodistally, the tooth movement trends were relatively not obvious. In terms of displacement magnitude for individual teeth, under different periodontal conditions and intrusion increments, the lateral incisors showed greater vertical intrusion than the central incisors. In contrast, lingual crown and labial root tipping was substantially more pronounced in the central incisors than in the lateral incisors and canines. For a given intrusion increment, the vertical and labiolingual displacements of both the incisors and canines increased progressively with greater alveolar bone loss (Figure 3). Under different periodontal conditions, tooth displacement showed the aforementioned increasing trend as the intrusion increment increased. With either increased alveolar bone loss or larger intrusion increments, the increased displacement in the lateral incisors primarily manifested as vertical intrusion. In contrast, for the central incisors, it predominantly manifested as lingual crown and labial root tipping (Figure 4). Figure 5 illustrates the absolute intrusion efficiency of the incisors under different periodontal conditions and intrusion increments. Given identical periodontal conditions and increments, the lateral incisor exhibited higher intrusion efficiency than the central incisor. For a constant increment, the intrusion efficiency of both teeth increased as alveolar bone loss progressed. Under the same periodontal condition, the intrusion efficiency of the central incisor increased with a larger increment, reaching a maximum of 27.3% in the M3 state with a 0.20-mm increment. Conversely, for the lateral incisor in the M2 and M3 states, a 0.15-mm increment yielded a higher intrusion efficiency than a 0.20-mm increment. Notably, the absolute intrusion efficiency of the lateral incisor peaked at 92.0% in the M3 state with a 0.15-mm increment. The displacement patterns of the CAs are presented in the Supplementary Material. Across all nine models, the aligner segment encompassing the four incisors showed an extrusive tendency, which was more pronounced at the central incisors than at the lateral incisors. This extrusive trend intensified with increasing designed intrusion and alveolar bone resorption.

**FIGURE 3:**
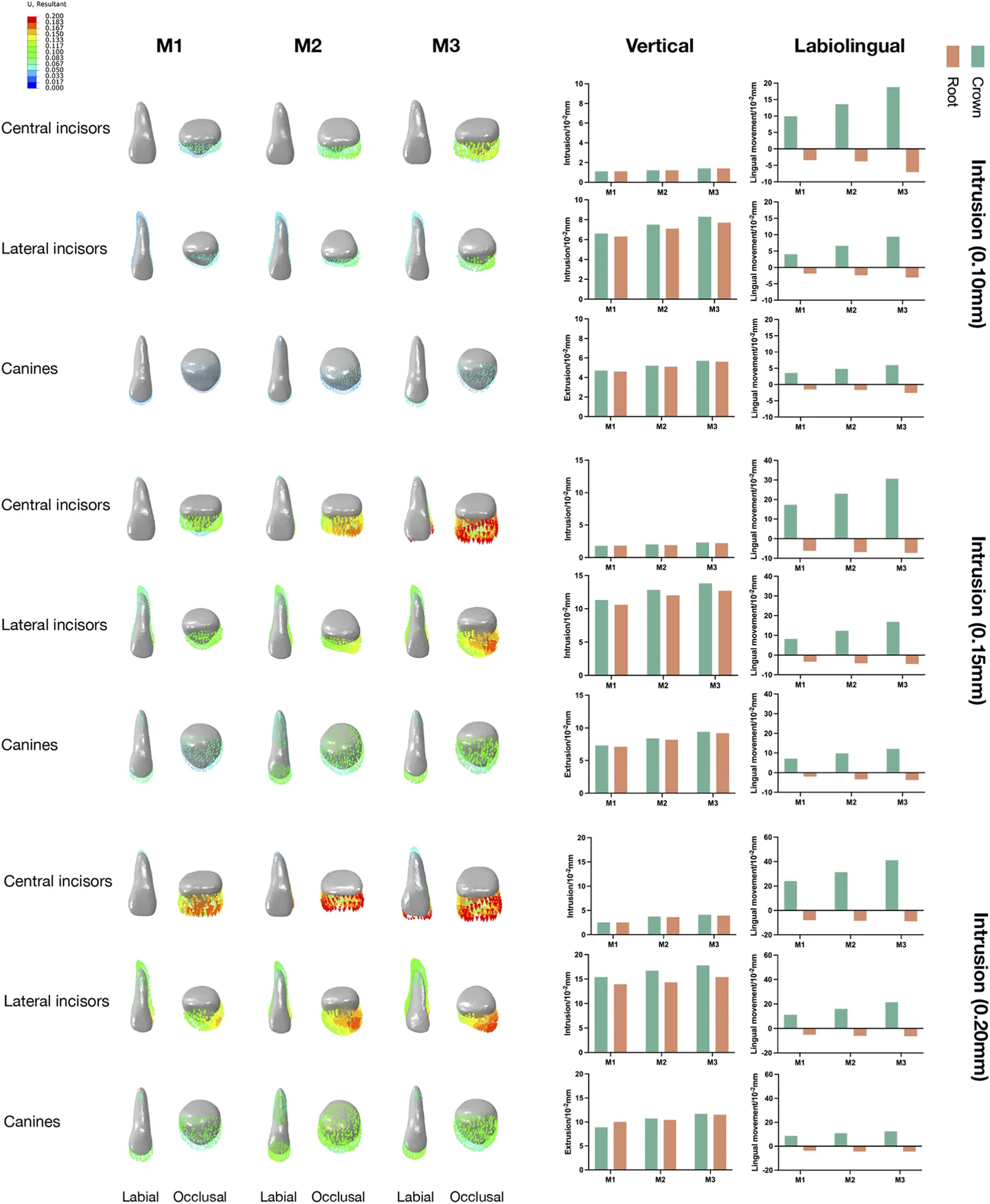
Displacement trends of the maxillary anterior teeth under three periodontal conditions. M1: Normal alveolar bone level; M2: One-third alveolar bone loss; M3: One-half alveolar bone loss.

**FIGURE 4:**
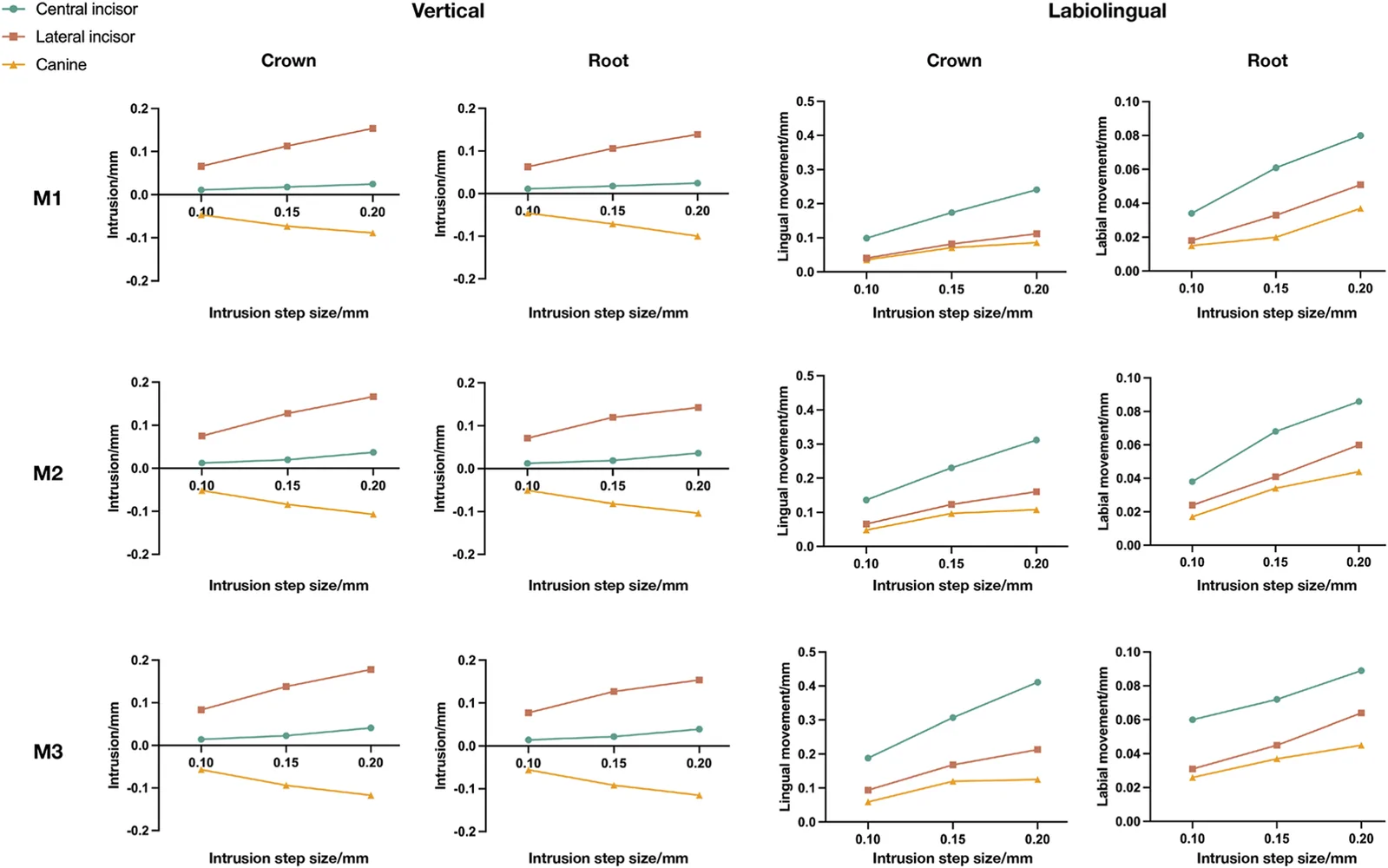
Vertical and labiolingual displacements of the maxillary anterior teeth at different intrusion increments under three periodontal conditions. M1: Normal alveolar bone level; M2: One-third alveolar bone loss; M3: One-half alveolar bone loss.

**FIGURE 5:**
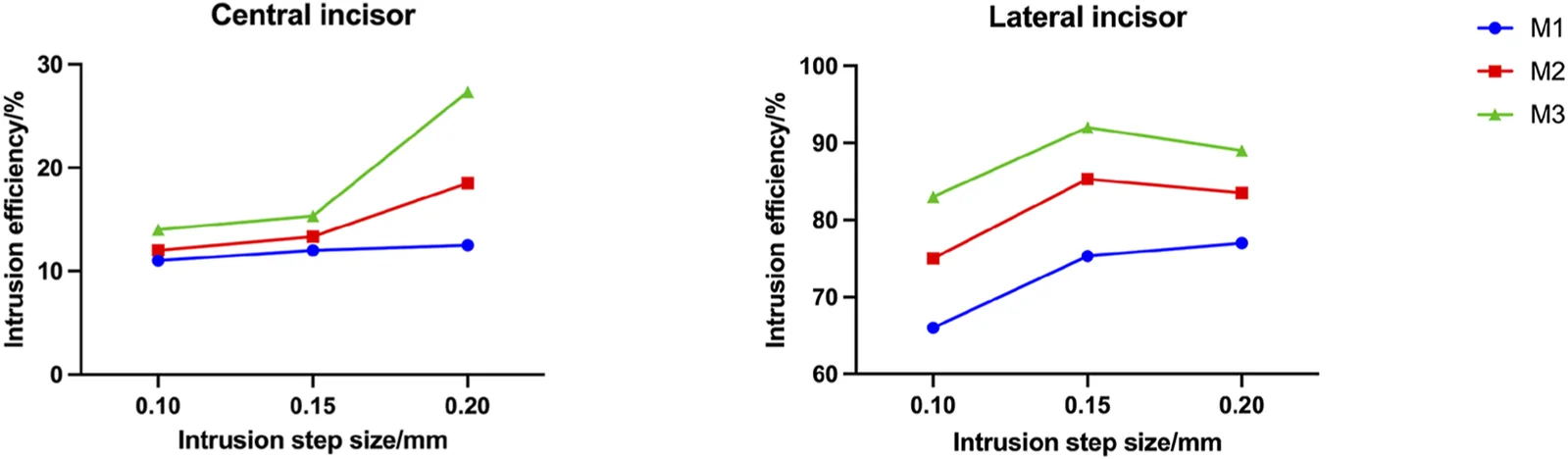
Absolute intrusion efficiency of the central and lateral incisors. M1: Normal alveolar bone level; M2: One-third alveolar bone loss; M3: One-half alveolar bone loss.

To further investigate the potential risks during incisor intrusion, von Mises stress in the roots, along with the maximum compressive stress within the PDL, was analyzed. Von Mises stress distribution showed peak values in two distinct regions: the incisal portion of the crown and the lingual aspect of the root. The extent of stress concentration progressively expanded with increasing intrusion increment. Under identical periodontal conditions and increments, the von Mises stress on the lateral incisor root was higher than that on the central incisor. Both increased alveolar bone loss and larger intrusion increments led to elevated von Mises stress on the incisor roots. In the M3 state with a 0.20-mm increment, the von Mises stress on the lateral incisor root (8.18 MPa) was approximately 2.8 times that observed with a 0.10-mm increment (2.96 MPa) (Figure 6).

**FIGURE 6:**
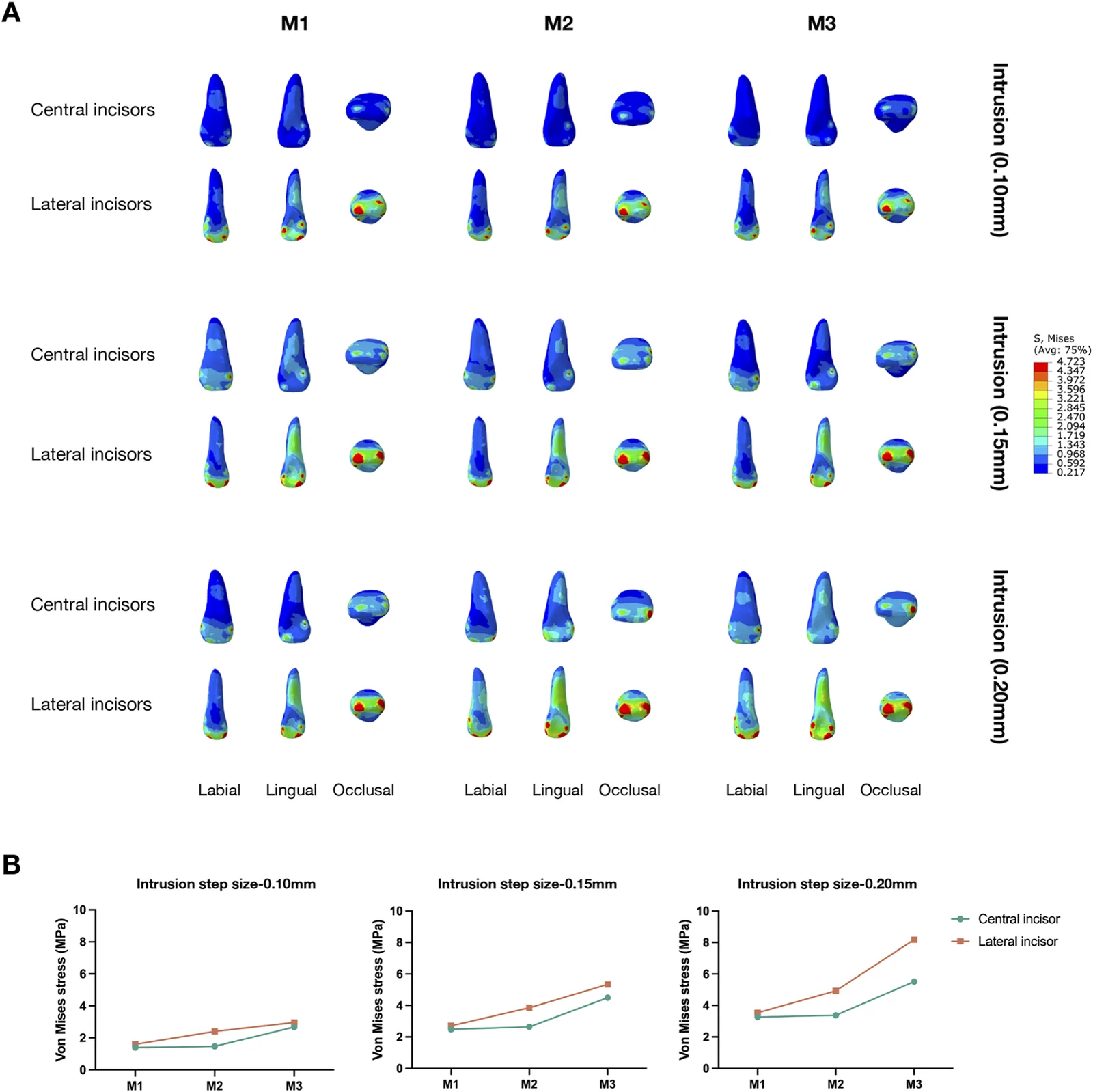
Von Mises stress of the central and lateral incisors under three periodontal conditions. **(A)** Von Mises stress distribution of the central and lateral incisors. **(B)** Von Mises stress values on the roots of the central and lateral incisors at different intrusion increments. M1: Normal alveolar bone level; M2: One-third alveolar bone loss; M3: One-half alveolar bone loss.

The maximum compressive stress distribution within the incisor PDL is presented in Figure 7A. Compressive stress concentrated predominantly at the labial apex and lingual cervical region. Increased alveolar bone loss and larger intrusion increments both resulted in higher maximum compressive stress values in the incisor PDL as well as a broader distribution of stress concentration zones. The lateral incisor PDL exhibited higher compressive stress than the central incisor. Under identical periodontal conditions and increments, the lateral incisor PDL sustained maximum compressive stress values approximately double those observed in the central incisor. In the M3 state with a 0.20-mm increment, the maximum compressive stress in the lateral incisor PDL (0.114 MPa) was approximately twice that observed with a 0.10-mm increment (0.056 MPa) (Figure 7B).

**FIGURE 7:**
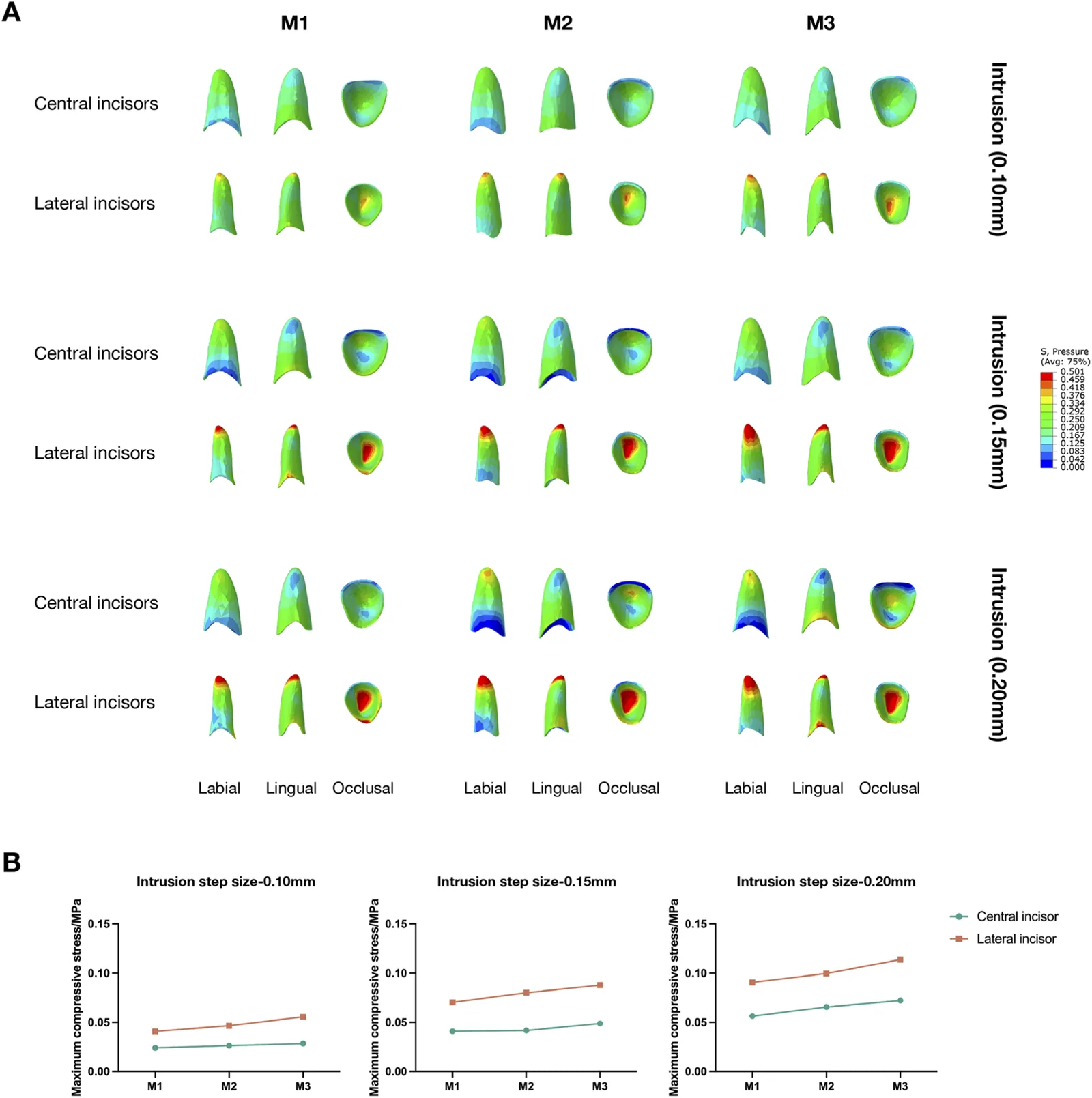
Maximum compressive stress in the periodontal ligament (PDL) of the central and lateral incisors under three periodontal conditions. **(A)** Maximum compressive stress distribution in the PDL of the central and lateral incisors. **(B)** Maximum compressive stress values in the PDL of the central and lateral incisors at different intrusion increments. M1: Normal alveolar bone level; M2: One-third alveolar bone loss; M3: One-half alveolar bone loss.

## 4 Discussion

On the basis of the 2018 periodontal disease classification, this research constructed finite element models of three periodontal conditions to simulate maxillary incisor intrusion using CAs in patients with different stages of periodontitis (Caton et al., 2018). Three different intrusion increments were designed to explore appropriate treatment protocols for different periodontal conditions. Our results showed that in all models, the central and lateral incisors achieved intrusion along their long axes, while the canines serving as anchorage teeth exhibited an extrusion tendency despite the addition of attachments, consistent with previous findings (Liu and Hu, 2018; Li et al., 2023). However, bodily intrusion of the incisors was not achieved; alongside intrusion, the incisors demonstrated obvious tipping, characterized by lingual crown displacement and labial root movement. This explains the clinical observation where incisors designed to intrude along their long axes paradoxically appear extruded perpendicular to the occlusal plane. Kregier et al. reported similar clinical findings, noting the underperformance of CAs in resolving anterior deep overbite (Krieger et al., 2012). Furthermore, these results highlight the critical need for torque control during maxillary incisor intrusion with CAs. Without adequate clinical control, this tendency may lead to labial bone fenestrations in the maxillary anterior region. When necessary, clinicians should consider incorporating power ridges into the gingival portion of the aligners or programming overcorrection for the incisors in their final positions.

Al-Balaa et al. used CBCT to evaluate the planned versus achieved anterior intrusion with Invisalign, finding a higher intrusion achievement rate for maxillary lateral incisors than for central incisors (Al-Balaa et al., 2021). Under different periodontal conditions and intrusion increments, our results also demonstrated a greater intrusion amount for lateral incisors than for central incisors, whereas central incisors exhibited a more pronounced tendency for lingual crown and labial root tipping. Consistent with this finding, the extrusive tendency of the CA segment covering the central incisors exceeded that observed at the lateral incisors, indicating a greater propensity for loss of aligner-tooth fitness at the central incisors. We speculate this is because the central incisors are further from the anchorage teeth; consequently, due to the material properties of CAs, the intrusive effect and mechanical control on the central incisors are diminished. Furthermore, the larger periodontal ligament area of the central incisors compared to the lateral incisors may render them more difficult to intrude under the same increment. In contrast, Kravitz et al. compared predicted and actual models and reported an average anterior intrusion efficacy of 41.3% for CAs, with maxillary lateral incisors showing the lowest predictability of only 33% (Kravitz et al., 2009). We attribute the discrepancy between their findings and ours to two possible factors. First, variations in tooth movement plans designed by different clinicians can lead to differences in actual clinical efficacy. Second, maxillary lateral incisors may respond more readily to intrusion, potentially causing a gap between the incisal edge and the aligner. This loss of tracking compromises the aligner-tooth fit, making it difficult to further express tooth movement in later stages. Regarding the off-track of CAs, no attachments were designed on the maxillary anterior teeth in this study. The extrusive tendency of the CAs combined with tooth intrusion may compromise the fit between the CAs and the dentition, thereby preventing the CAs from delivering sufficient intrusive force to achieve effective intrusion. A recent finite element study on maxillary anterior tooth intrusion demonstrated that the addition of attachments in the anterior region facilitated intrusion and correction of overbite (Shah et al., 2026). However, in periodontitis patients with alveolar bone resorption, excessive attachment placement increases the force required for aligner insertion and removal, which may exacerbate tooth mobility. Therefore, in clinical practice, attachment design should be carefully balanced against periodontal status and the efficiency of tooth movement.

As alveolar bone height decreases, teeth become more susceptible to movement under the same orthodontic force. Furthermore, single-rooted teeth exhibit increased tipping tendency due to apical migration of their center of resistance (Frias Cortez et al., 2022). Our results confirmed this phenomenon: under the same increments, maximum 3D tooth movement and tipping tendency were observed in the M3 group. This indicates that during maxillary incisor intrusion with CAs, periodontitis patients with alveolar bone loss may face a higher risk of anterior bone fenestrations, warranting close clinical monitoring. We further calculated the absolute intrusion efficiency of the incisors under different periodontal conditions and intrusion increments. Central incisor data demonstrated that both the deterioration of the periodontal condition and an increase in the intrusion increment increased their absolute intrusion efficiency. Conversely, for lateral incisors in the M2 and M3 states (representing one-third and one-half alveolar bone loss, respectively), the intrusion efficiency with a 0.2-mm increment was paradoxically lower than that with a 0.15-mm increment. We attribute this finding to several factors: reduced alveolar bone height increases tooth mobility, and lateral incisors, being closer to the anchorage teeth, express intrusive movements more readily. However, given their smaller crowns, a 0.2-mm activation increment may produce incisal edge discrepancies between the aligner and tooth, resulting in tracking loss. In contrast, a 0.15-mm increment likely maintains superior aligner-tooth adaptation, resulting in more efficient force transmission.

Because maxillary anterior intrusion often leads to root resorption, root von Mises stresses and maximum PDL compressive stresses were additionally assessed for the incisors (Zheng et al., 2026). The results indicated that for dental hard tissues, von Mises stresses were predominantly distributed along the incisal edges and lingual root aspects. For the PDL, maximum compressive stresses were largely concentrated at the apex and lingual cervical margin. Under the same periodontal conditions and intrusion increments, lateral incisors showed higher root and PDL stresses than central incisors, in agreement with the results reported by Li et al. (2023). This implies that compared to central incisors, lateral incisors are more prone to root resorption and lingual bone dehiscence during CA intrusion. Under the same intrusion increments, as the periodontal condition deteriorated, the areas of stress concentration on the roots and PDL expanded, and the stress values increased. With one-half alveolar bone loss, the lateral incisor root von Mises stress at a 0.2-mm increment was 2.8 times that at 0.1 mm, with maximum PDL compressive stress doubling correspondingly. Excessive stress increases the susceptibility to root resorption and may further contribute to tooth mobility and alveolar bone loss in periodontitis patients with reduced metabolic activity. Based on the stress analysis and intrusion efficiency outcomes of this study, we recommend that the incisor intrusion increment not exceed 0.15 mm when a patient has more than one-third alveolar bone loss. Beyond reducing the planned increment, safe orthodontic therapy for periodontitis patients during maxillary incisor intrusion can also be achieved by extending the wear time of each aligner and selecting aligner materials with a lower elastic modulus (Li et al., 2023; Zhong et al., 2025).

Several limitations should be acknowledged. First, the periodontitis models were constructed based on the 2018 classification using a single volunteer model with normal occlusion. While this eliminated confounding variables from individual anatomical variations, the results may not fully apply to patients with diverse clinical presentations. For instance, a previous 3D FE study on mandibular anterior intrusion revealed that the incisor mandibular plane angle (IMPA) variations affect intrusion behavior (Li et al., 2023). Second, the model established in this study was static in nature, capturing only the instantaneous deformation and stress distribution immediately after loading, without simulating the biological remodeling process associated with orthodontic tooth movement (Chen et al., 2026). Regarding material properties, the PDL was modeled using simplified assumptions that did not account for its time-dependent and viscoelastic behavior, although prior studies have suggested that such simplification is acceptable when deformation magnitudes are small (Qian et al., 2001; Hemanth et al., 2015a; Hemanth et al., 2015b; Shah et al., 2026). Besides, CAs in FE studies are typically modeled with uniform thicknesses of 0.45–0.75 mm (Yokoi et al., 2019; Gao et al., 2024; Li et al., 2024). In reality, aligners demonstrate thickness variability in clinical applications. Furthermore, the simulated intrusion in this study was directed along the long axes of the incisors. Intrusion directed away from the long axis may yield different outcomes. Recently, Su et al. demonstrated through FEA that, compared with intrusion along the long axis of the tooth, an angulated intrusion design appears to mitigate the tipping movement associated with maxillary anterior tooth intrusion (Su et al., 2026). Consequently, FEA cannot perfectly replicate actual clinical scenarios. Further clinical investigations are required to corroborate these findings and provide more accurate and reliable conclusions. Nonetheless, the tooth displacement trends and PDL stress patterns derived from this FEA provide predictive insights into maxillary incisor intrusion under different periodontal conditions.

## 5 Conclusion

1. Intrusion of maxillary incisors along their long axis using CAs achieves intrusion, accompanied by a tendency for lingual crown and labial root tipping. Lateral incisors showed higher absolute intrusion efficiency and endure greater root and PDL stresses than central incisors.
2. Alveolar bone loss exacerbates both the tipping tendency and the stresses exerted on the roots and PDL during intrusion; therefore, careful biomechanical design is essential to ensure safe and efficient treatment. Specifically, when alveolar bone loss exceeds one-third, the aligner intrusion increment is recommended to be limited to a maximum of 0.15 mm.

## Data Availability

The original contributions presented in the study are included in the article/Supplementary Material, further inquiries can be directed to the corresponding author.
