# Supplementary material for: Biomechanical analysis of maxillary incisor intrusion with clear aligners under different periodontal conditions: a finite element analysis
Source: Front Bioeng Biotechnol. 2026 Sep 23;14:1921947. doi: 10.3389/fbioe.2026.1921947 (PMC13642637; doi:10.3389/fbioe.2026.1921947)
Supplement: Supplementary file 1 [file Supplementaryfile1.docx]

Supplementary material. The displacement of clear aligners.
